# Interferon-α Subtypes As an Adjunct Therapeutic Approach for Human Immunodeficiency Virus Functional Cure

**DOI:** 10.3389/fimmu.2018.00299

**Published:** 2018-02-22

**Authors:** Jeffy George, Joseph J. Mattapallil

**Affiliations:** ^1^Uniformed Services University, Bethesda, MD, United States

**Keywords:** human immunodeficiency virus, functional cure, interferon-α, interferon-α subtypes, human immunodeficiency virus latency

## Abstract

Human immunodeficiency virus (HIV) establishes life-long latency in infected individuals. Although highly active antiretroviral therapy (HAART) has had a significant impact on the course of HIV infection leading to a better long-term outcome, the pool of latent reservoir remains substantial even under HAART. Numerous approaches have been under development with the goal of eradicating the latent HIV reservoir though with limited success. Approaches that combine immune-mediated control of HIV to activate both the innate and the adaptive immune system under suppressive therapy along with “shock and kill” drugs may lead to a better control of the reactivated virus. Interferon-α (IFN-α) is an innate cytokine that has been shown to activate intracellular defenses capable of restricting and controlling HIV. IFN-α, however, harbors numerous functional subtypes that have been reported to display different binding affinities and potency. Recent studies have suggested that certain subtypes such as IFN-α8 and IFN-α14 have potent anti-HIV activity with little or no immune activation, whereas other subtypes such as IFN-α4, IFN-α5, and IFN-α14 activate NK cells. Could these subtypes be used in combination with other strategies to reduce the latent viral reservoir? Here, we review the role of IFN-α subtypes in HIV infection and discuss the possibility that certain subtypes could be potential adjuncts to a “shock and kill” or therapeutic vaccination strategy leading to better control of the latent reservoir and subsequent functional cure.

## Introduction

Human immunodeficiency virus (HIV) infections are characterized by severe immunodeficiency and onset of opportunistic infections. Currently, there are over 36 million people worldwide who are living with HIV. Onset of highly active antiretroviral therapy (HAART) has led to better viral control and long-term outcome in HIV-infected patients. As access to therapy becomes more readily available around the world, the number of new infections and transmission are expected to dramatically decrease, raising the hope that the HIV epidemic can be controlled and managed. Encouraging studies (1) showing the efficacy of neutralizing antibodies to control viral rebound and the development of long-lasting drugs are likely to have a major impact on the epidemiology of the disease. As major efforts to control the HIV epidemic gets underway, focus has shifted to finding cure for patients who are already infected with HIV.

Human immunodeficiency virus is a retrovirus that integrates into the host genome. As such, an HIV-infected individual is infected for life. The primary target cell for HIV is the CD4 T cell, with HIV establishing latency in these cells, and this latent reservoir continues to persist during HAART. Except in the case of Timothy Brown who is the only known case of HIV to have been completely cured, complete eradication of HIV reservoir has proven to be challenging not only due to the integration of HIV into the host genome but also due to the large size of the latent persistent reservoir. As such focus has recently shifted to the development of functional cure strategies, where the objective is to obtain complete remission in the absence of antiretroviral drugs.

Evidence for functional cure came rather serendipitously when an infant born to an HIV-infected mother was treated continuously for over 2 years within hours after birth. The child remained free of HIV for about 2 years after withdrawal of therapy raising the prospect that early HAART could potentially achieve full remission in HIV-infected subjects. However, the excitement was short lived as HIV rebounded suggesting the latent reservoir was not eradicated with early therapy and reactivated in the absence of long-term HAART. A number of novel approaches such as “shock and kill” using latency reversing agents (LRA) although somewhat successful in reactivating latent HIV (1), their impact on the viral reservoir has been rather limited, suggesting that LRA would need to be combined with other approaches such as vaccination against HIV that can simultaneously activate the immune system to recognize viral antigens expressed on the surface of latently infected cells following reactivation with LRA. A number of studies are currently underway to explore this strategy.

Other strategies have focused on activating intracellular defense mechanisms using interferon α (IFN-α) in combination with LRA or other immune mediators with some promising data from non-human primate models. Here, we review the progress that has been made to date in understanding the role IFN-α plays in HIV infection and explore the potential for harnessing IFN-α and its subtype as a strategy toward functional cure.

## Type I IFN and HIV Infection

Since its initial discovery in 1957 as factors that inhibit viral replication (2), the role of innate IFN in viral infections has been extensively studied. The primary source of IFN-α is the plasmacytoid DC (pDC), whereas IFN-β is produced by most cell types (3, 4). pDC plays a major role in regulating the immune system and are the earliest cells recruited to the sites of virus entry. In response to viral pathogen-associated molecular patterns, pDCs have been shown to produce ~1,000-fold more IFN-α/β than other cell types (5).

Plasmacytoid DC express a variety of pathogen recognition receptors (PRRs) such a Toll-like receptor (TLR) 3, TLR7, TLR8, and TLR9 that can sense viral nucleic acids leading to the secretion of IFN-α (6–8). Recent studies have demonstrated that the cytoplasmic DNA sensor cGAS plays an important role in the secretion of IFN-α during both HIV and SIV infections (9). Lahaye et al. (10) showed that DC’s sense viral cDNA in the cytoplasm that was mediated by cGAS and blocking cGAS or reverse transcription inhibited these responses (11). Likewise, Herzner et al. (12) showed that single-stranded HIV-1 DNA activates cGAS and HIV-1 reverse transcripts was the predominant viral DNA found in the cytoplasm during early infection. George et al. (3) showed that treatment with reverse transcriptase inhibitors immediately after infection completely blocked plasma IFN-α in SIV-infected rhesus macaques. Taken together these studies show that numerous innate sensing PRR contribute to the induction of IFN-α responses during HIV infection.

Although the production of IFN-α during HIV infection has been clearly demonstrated, the exact role these IFN play during infection has been less clear. Blockade of IFN-αR with anti-IFN-αR antibody was associated with higher HIV replication, whereas HIV replicated at lower levels in pDC-depleted cultures treated with IFN-α (13). IFN-α was found to limit HIV-1 replication by decreasing the formation of late reverse transcriptase products in infected cells (14), and treatment of newly infected CD4 T cells with IFN-α for short period time was associated with significant inactivation of HIV during the early stages of replication (15). IFN-α was shown to slow HIV disease progression in randomized, placebo-controlled trials (16), and Asmuth et al. (17) reported that the treatment with pegylated IFN-α2a had a statistically significant anti-HIV effect. Others have shown that IFN-α treatment inhibited HIV and SIV replication in CD4 T-cell lines (18), monocytes (19), and macrophages (20). IFN-α has been reported to affect late stages of HIV-1 replication in chronically infected cells, by inhibiting virus assembly and release and reducing the infectivity of virions (21). Other studies have shown the IFN-α induced IFN-stimulated genes (ISGs) that effectively suppressed HIV replication (22–24).

Interferon α has been shown to induce numerous ISG that are capable of restricting HIV replication namely, apolipoprotein B mRNA-editing (APOBEC3) family of cytidine deaminases, TRIM5α, tetherin (BST-2), SAMHD1, MX2, etc. (25–27). Studies have reported high levels of ISG expression in CD4 T cells very early during infection (28), and increased levels of APOBEC3G was found to correlate with lower levels of infection in macrophages during SIV infection (29). Others have reported that ISG were significantly upregulated during SIV infection (30–33). In addition to the induction of ISG, IFN-α has been shown to prime adaptive immune responses by cross-presenting viral antigens to CD8^+^ T cells (34–36). Interestingly, Boasso et al. demonstrated that IFN-α-induced indoleamine 2,3-dioxygenase (IDO) from pDC inhibited CD4^+^ T-cell proliferation during HIV infection (37), and blockade of gp120/CD4 interactions was found to inhibit HIV-mediated induction of IDO and IFN-α (38, 39).

In contrast to the protective effects of IFN-α during HIV infection, increased production of IFN-α was accompanied by an increase in HIV loads (40). Mandl et al. (41) argued that the generalized immune activation and progressive CD4 T cell depletion observed in pathogenic SIV infection was likely due to an aberrant activation of the innate immune system and increased IFN-α production in contrast to natural hosts such as sooty mangabeys. Martinson et al. (42) reported that TLR stimulation and IFN-α secretion by pDC contribute to immune activation during HIV infection. Others have shown that rapid progression of HIV was associated with continuous production of IFN-α, likely through enhanced T cell differentiation and activation (43). Parrish et al (44) demonstrated that transmitted founder viruses replicate and spread more efficiently in CD4 T cells in the presence of IFN-α. Fraietta et al. (45) showed that IFNα/β upregulated the expression of Bak, a pro-apoptotic protein that correlated with increased T cell apoptosis, low CD4^+^ T cell counts and high viral loads in HIV-infected patients. Patients who progressed to disease were found to have lower levels of pDC but displayed higher levels of IFN-α and MxA compared to healthy individuals (46). Other studies have reported that IFN-α promoted chronic immune activation, apoptosis, and immune dysfunction during HIV-1 infection (47–51). Likewise IFN-α was found to regulate CD4^+^ T-cell apoptosis induced by noninfectious HIV-1 by upregulating the expression of TNF-related apoptosis-inducing ligand (TRAIL) (38). Cha et al. (52) reported that IFN-α significantly enhanced activation-induced proliferation but not homeostatic proliferation, suggesting that the IFN-α likely promotes the loss of CD4 T cells by accelerating cell turnover and activation-induced cell death. On the other hand, Dondi et al. found that IFN-α displays contrasting proliferation-inducing and proapoptotic properties (53). Chronic IFN-α signaling has been implicated in other persistent viral infections such as LCMV (54, 55).

## Type I IFN Subtypes and HIV Infection

Since its initial discovery, numerous isoforms of type I IFN have been identified. These isoforms, encoded by single exon genes include IFN-α (which harbors 13 different subtypes namely, IFN-α1, α2, α4, α5, α6, α7, α8, α10, α13, α14, α16, α17, and α21), IFN-β, IFN-ε, IFN-κ, and IFN-ω (56). All the type I IFN subtypes signal through a common receptor complex consisting of IFN-αR1 and IFN-αR2 subunits. In humans, IFN-α subtypes share ~70–99% amino acid sequence identity with each other and a ~35% identity with IFN-β (57).

The evolutionary advantage of having multiple isoforms of the same gene that bind to a common receptor complex is not clear. However, there is evidence that the different subtypes display variable binding affinities for the common receptors (58–60), which in turn appears to influence their efficacy and potency (summarized in Table 1). Subtypes such as IFNα-10 binds to the IFN-αR1/2 receptor complex at affinities that is 10- to 100-fold greater than IFNα-1 (61). Interestingly, IFNα-10 was found to be highly effective against Semliki forest virus and Vesicular stomatitis virus, whereas IFN-α1 was the least effective among the nine different subtypes tested (61). Cull et al. (62) examined the expression of IFN-α1, α2, α4, α5, α6, and α9 and IFN-β in murine cytomegalovirus-induced myocarditis and observed that IFN-α6 reduced viral replication and inflammation in contrast to IFN-α2 and α5 that increased replication.

**Table 1:** Antiviral activity of IFN-α subtypes.

IFN subtype(s)	Viral infection	Effect	Reference
IFN-α1, 4, and 9	MCMV	IFN-α1 transgene showed better antiviral activity than IFN-α4 or IFN-α9	Yeow et al. (63)
IFN-α1, 2, 4, 5, 6, and 9 and IFN-β	MCMV	IFN-α6 transgene reduced MCMV replication, whereas IFN-α5 increased viral replication	Cull et al. (62)
IFN-α1, 2, 5, 8, 10, 14, 17, and 21 and IFN-β	MEV	IFN-α 5, 8, 10, 14, and 17 were highly effective, whereas IFN-α2 had a moderate effect and IFN-α1 was least effective	Foster et al. (64)
IFN-α1, 2, 5, and 8 and 10	HCV	IFN-α8 was effective in suppressing HCV replication, whereas IFN-α1 is least effective	Koyama et al. (65)
IFN-α2, 6, 8, and 14 and IFN-β	HIV	Plasmids encoding IFN-α2, 6, 8, and 14 and IFN-β showed IFN-α14 and IFN-β were more protective than other subtypes in humanized mice	Abraham et al. (66)
IFNα4 and IFNα5	HBV	Both proteins and plasmid encoding IFN-α4 and 5 showed anti-HBV activity	Song et al. (67)
IFN-α1, 2b, and 4b	Influenza A virus	IFN-α2b showed strong antiviral activity as compared to IFN-α1 or 4b	Moll et al. (68)
IFN-α1, 2, 5, 6, 7, 8, 10, 14, 17, and 21	hMPV	IFN-α5, 6, 8, and 10 had higher antiviral activity	Scagnolari et al. (69)
IFN-α2 and 14	HIV (humanized mice)	IFN-α14 suppressed HIV replication, induced tetherin, MX2, APOBEC3G, and increased numbers of TRAIL + NK cells compared to IFN-α2	Lavender et al. (70)
IFN-α1, 2, 4, 6, 8, 14, 17, and 21	MuV	IFN-α6 showed higher antiviral activity	Markusic et al. (71)
IFN α-01/13, 2, 6, 8, 14, 16, 23, 24, 25, 26, 27, 28, and 29, IFN-β, IFN-ω, and IFN-λ1	SIV	IFN-α01/13, 2, 6, 8, 14, 16, 23, 24, 25, 26, 27, 28, 29, IFN-β, IFN-ω, and IFN-λ1 were significantly increased in lymph nodes at day 10 postinfection compared to restricted expression in PBMC (IFN-α01/13 and IFN-λ1) and jejunum (IFN-α1, 6, 8, 14, and 23, IFN-ω, and IFN-λ1). Primary source of all subtypes were dendritic cells (DC)	George et al. (3)
Pegylated IFN-α	HIV	Treatment with pegIFN-α and ribavirin reduced HIV DNA and increased frequencies of NK cells in HIV-1/HCV-infected patients	Hua et al. (90)

*HIV, human immunodeficiency virus; hMPV, human metapneumovirus; IFN, interferon; MEV, murine encephalomyelitis virus; MuV, mumps virus; TRAIL, TNF-related apoptosis-inducing ligand*.

Sperber et al showed that IFNα-2 induced chemotaxis genes and was most effective against HIV-1 (72) whereas IFN-α8 induced ISG’s that were protective against HCV replication (65). Foster et al (64) showed that IFN-α8 has very high antiviral potency compared to some of the other subtypes. On the other hand, Scagnolari et al. (69) reported that IFN-α5, 6, 8, and 10 had high potency against human metapneumovirus, whereas IFN-α2, 17, and 21 were the least potent. Others have shown significant differences in the *in vitro* antiviral and antiproliferative effects of various subtypes (73–75). Hibbert and Foster (76) examined the effect of various subtypes on human B cells and showed that IFN-α8 induced proliferation at very low concentrations compared to other subtypes with IFN-α1 being largely inactive. Likewise, Hilkens et al. (77) examined the signaling though Janus kinase/STAT and transcriptional responses to selected IFN-α subtypes in human T cells and dendritic cells and reported differences in the potency of various subtypes to induce ISG.

Numerous studies have examined the expression of IFN subtypes during both HIV and SIV infections. Zaritsky et al. (78) evaluated the expression of both total IFNα mRNA and the pattern of IFN-α subtype mRNA expression in macaques during acute SIV infection and found that all 13 subtypes were expressed in the spleen with IFN-α4, 17, and 21 being the least abundant as compared to high levels of IFN-α2, 8, and 13. In contrast, only subtypes IFN-α2, 6, and 13 were expressed in the brain, whereas subtypes IFN-α6 and 13 were upregulated in the lung suggesting to tissue-specific differences in the expression of various subtypes. Lehman et al. (46) reported that IFN-α2 and IFN-α6 were significantly upregulated in HIV-infected patients. On the other hand, Li et al. (79) showed that IFN-α2 and 16 were upregulated during chronic HIV infection. George et al. (3) examined the expression of both type I and III IFN subtypes in peripheral blood, jejunal mucosa, and lymph nodes (LNs) of SIV-infected rhesus macaques and reported that all subtypes (IFN-α01/13, 02, 06, 08, 14, 16, 23, 24, 25, 27, 28, and 29, IFN-β, IFN-ω, and IFN-λ1) were significantly elevated in the LNs at day 10 postinfection compared to a restricted expression in PBMC (IFN-α01/13 and IFN-λ1) and jejunal mucosa (IFN-α1, 6, 8, 14, and 23, IFN-ω, and IFN-λ). Harper et al. (80) evaluated the expression of different IFN-α subtypes and their potency in HIV-1-exposed pDC using the lamina propria aggregate *ex vivo* culture model and reported that HIV infection induced numerous IFN-α subtypes with IFN-α6, IFN-α8, and IFN-α14 being the most potent at inhibiting HIV infection. Earlier studies (72) have shown that IFN-α2 was effective at suppressing HIV-1 replication although more recent studies (70) have demonstrated that IFN-α14 displayed significantly higher antiviral activity than IFN-α2 against HIV infection in humanized mouse models.

## IFN-α Subtypes and Potential for Functional Cure

Given the potential for IFN-α to induce immune activation during HIV infection, there is a potential concern regarding its use in functional cure strategies although there is anecdotal evidence that IFN-α could suppress viral replication during antiretroviral therapy.

Treatment of HIV-infected subjects under HAART with pegylated-IFN-α2a was associated with the suppression of HIV RNA loads (81). Likewise, Sun et al. (82) demonstrated that the treatment of HIV/HCV co-infected patients with IFN-α/ribavirin during HAART led to a moderate but significant and sustained decline in cell-associated HIV DNA. Recent reports using IFN-α in combination with other factors appear promising. Micci et al. (83) reported that a combination of recombinant IL-21 and pegylated-IFN-α2a limited residual inflammation and viral persistence in SIV-infected rhesus macaques and significantly delayed viral rebound after withdrawal of antiretroviral therapy. Others (84) have shown that pretreatment of CD4 T cells with IFN-α and IFN-β reversed HIV latency in T-cells both *in vitro* and *ex vivo* and was associated with a reduction in the number of latently infected cells. Azzoni et al. (81) demonstrated that pegylated-IFN-α2 monotherapy successfully suppressed HIV-1 replication and reduced cell-associated HIV DNA.

Recent studies by Lavender et al. (70) showed that IFN-α14 when delivered at the same clinical dose as IFN-α2 to humanized mice significantly suppressed HIV replication and proviral loads and reduced immune activation that was accompanied by induction of high levels of APOBEC3G, MX2, and tetherin that have been shown to interfere with HIV replication (85–88). Abraham et al. (66) showed that gene therapy with plasmids encoding IFN-β and IFN-α14 significantly suppressed HIV-1 replication in mice for longer periods of time compared to other commonly used subtypes such as IFN-α2. Interestingly, all treated mice rebounded after cessation of IFN-α14 treatment. Additional studies are warranted to determine if the protective efficacy of IFN-α14 activated specific innate defenses during antiretroviral therapy that were different from those induced by other subtypes tested. These studies, however, raise the possibility that IFN subtypes such as IFN-α14 could be a potent adjunct to current approaches exploring functional cure strategies in HIV-infected subjects.

Other studies have shown that specific IFN subtypes were more potent at activating NK cells that could be harnessed to eradiate latently infected cells after reactivation. Gibbert et al. (89) demonstrated that IFN-α11-activated NK cells that enabled cytolytic killing of Friend retrovirus-infected cells compared to other subtypes such as IFN-α2 and IFN-α5. Hua et al. (90) recently reported that the treatment of HIV-1/HCV co-infected subjects on HAART with pegylated-IFN-α induced activation of CD56^bright^CD16^−^ and CD56^bright^CD16^+^ NK cells expressing NKG2D an NKp30 that significantly correlated with a decrease in level of HIV-1 viral reservoir in CD4 T cells. Song et al. (67) examined that the effect of IFN-α subtypes on HBV infection and found that IFN-α4 and IFN-α5 correlated with expansion of effector NK cells in both liver and spleen that was associated with better control of HBV replication. Treatment of HIV-infected humanized mice with IFN-α14 was found to increase the expression of cytotoxic molecule TRAIL in NK cells, whereas Stegmann et al. (91) showed that induction of TRAIL on NK cells by IFN-α was associated with better control of hepatitis C infection. NK cells play an important role in the control of HIV infections (92) and strategies that can enhance NK cell activity could be beneficial in eradicating latently infected cells.

The studies described above suggest that a subset of IFN subtypes may be more effective at controlling infection than the others although there is a significant gap in our knowledge regarding the timing of administering these subtypes in the context of suppressive HAART that could potentially impact their efficacy. Sandler et al. (32) treated SIV-infected rhesus macaques with IFN-α2 during the acute phase of infection and reported that IFN-α2 initially upregulated the expression of antiviral genes, whereas continuous treatment was accompanied by desensitization and an increase in the viral reservoir size. Although the effect of initiating IFN therapy early in infection appears to be apparent, it is not clear if subjects under suppressive HAART regimens when treated would be unresponsive to treatment with various IFN subtypes.

Two exciting new studies (93, 94) using the humanized mouse model have reported that that blocking IFN signaling and reducing IFN-induced activation by treating with an antibody to the IFN receptor could reduce the size the HIV reservoir and delay viral rebound after cessation of HAART. These studies appear to be in contrast to what has been reported earlier using non-human primate models where blockade of IFN-αR was found to have the opposite effect (32). Audige et al. reported that treatment with anti-IFN-αR antibody was associated with increased HIV replication (13). On the other hand, blockade of chronic IFN signaling was shown to decrease immune activation and clear persistent LCMV infection in mice (55). Additional studies are needed to better clarify and confirm these findings in HIV infected subjects.

## Conclusion

Functional cure strategies that can eradicate the viral reservoir are urgently needed. A number of approaches are being currently explored to achieve this goal. Although IFN-α therapy has been attempted in the field, there is new evidence suggesting that specific subtypes such as IFN-α8 and 14 may display more potent efficacy against HIV infection than the subtypes such as IFN-α2 that have been used in the past. Whether these subtypes can enhance innate immune defense during suppressive HAART and if these innate defenses would be sufficient to eradicate the reactivated latent reservoir remains to be seen. Studies that use a combination of approaches such as specific IFN-α subtypes along with therapeutic immunization to activate both the innate and adaptive immune responses during suppressive HAART are likely to be more effective at achieving full remission of HIV.

## Author Contributions

All authors listed have made a substantial, direct, and intellectual contribution to the work and approved it for publication.

## Conflict of Interest Statement

The authors declare that the research was conducted in the absence of any commercial or financial relationships that could be construed as a potential conflict of interest.
